# Effects of HP-Guar Nano-emulsion Artificial Tears on Symptoms and Severity of Meibomian Gland Dysfunction in Evaporative Dry Eye During a 90-Day Longitudinal Study

**DOI:** 10.1007/s44402-026-00089-1

**Published:** 2026-04-27

**Authors:** Hugo Pena-Verdeal, Jacobo Garcia-Queiruga, Alba Castro-Giráldez, Belén Sabucedo-Villamarin, Carlos Garcia-Resua, Maria J. Giraldez, Eva Yebra-Pimentel

**Affiliations:** 1 GI-2092-Optometry, Departamento de Física Aplicada (Área de Optometría), Universidade de Santiago de Compostela, Campus Vida s/n, Santiago de Compostela, Spain; 2 Instituto de Investigación Sanitaria (IDIS), Santiago de Compostela, Spain

**Keywords:** Evaporative dry eye, HP-Guar nano-emulsion, Meibomian gland dysfunction, Ocular inflammation, Tear film stability

## Abstract

**Background:**

To evaluate whether hydroxypropyl-guar (HP-Guar) nano-emulsion artificial tears improve symptoms, quality of life, severity of meibomian gland dysfunction (MGD) and evaporative dry eye (EDE)-related parameters in mild-to-moderate EDE participants over 90 days.

**Methods:**

Fifty-one mild-to-moderate EDE participants were recruited for a single-masked, longitudinal clinical trial without a control group or placebo. Inclusion criteria comprised: Ocular Surface Disease Index (OSDI) between 13–32 points; at least one positive diagnostic sign (Non-Invasive Tear Break-Up Time [NIBUT] <10 s and/or corneal staining >1) and two classification signs (Tear Meniscus Height [TMH] ≥0.20 and ≤0.50 mm and MGD score ≥5 points). Participants applied 1–2 drops of HP-Guar nano-emulsion artificial tears per eye, four times daily for 90 days, with follow-up assessments at baseline, 30, 60 and 90 days. Three groups of tests were conducted each session: DED symptomatology and quality of life (OSDI, EuroQol 5-Dimension 5-Level [EQ-5D-5L]), MGD severity (MGD score, meibomian gland loss area [MGLA]) and EDE-related parameters (meibomian gland expression [MGE], Meibometry, lipid layer pattern (LLP), NIBUT, ocular redness and corneal staining).

**Results:**

Significant differences in OSDI score, EQ-5D-5L in the ‘Healthy today’ scale, MGD score, lower eyelid MGLA and LLP were observed across sessions (all *p* ≤ 0.03). However, no significant differences were found in EQ-5D-5L score, upper eyelid MGLA, MGE, Meibometry, NIBUT-first, NIBUT-average, limbal or bulbar redness in any area or corneal staining (all *p* ≥ 0.09).

**Conclusions:**

This 90‑day single‑masked study without a control group found that HP‑Guar nano‑emulsion eye drops were associated with improvements in symptoms, perceived health and MGD‑related signs. These findings are exploratory and require confirmation in controlled trials.

Key Points

- This study shows that a hydroxypropyl-guar nano‑emulsion tear formulation can meaningfully reduce everyday eye discomfort over 90ninety days, improving how individuals with evaporative dry eye feel in daily life.
- The tear formulation strengthened the outer tear film layer and supported eyelid gland health, indicating a potential role in slowing changes linked to long‑standing ocular surface problems.
- These findings suggest that tear formulations designed to stabilise the tear film surface may offer broader benefits than traditional drops, helping individuals maintain comfort and overall daily functioning.

## Introduction

Evaporative dry eye (EDE) is the most prevalent form of dry eye disease (DED) and is primarily associated with meibomian gland dysfunction (MGD), a chronic condition affecting the structure and function of the meibomian glands [1, 2]. These glands play a critical role in maintaining tear film stability by secreting lipids that reduce tear evaporation [3]. When glandular function is compromised due to phenomena such as obstruction, atrophy or altered lipid composition, tear film instability occurs, leading to increased evaporation, hyperosmolarity, ocular inflammation and epithelial damage [4]. Therefore, patients with EDE often experience persistent ocular discomfort, visual disturbances and a significant reduction in quality of life.

EDE originates from an underlying dysfunction of the eyelids. Therapeutic strategies proposed in the literature have not only focused on symptom relief but also on restoring the structural and functional integrity of the meibomian glands. Current management approaches include artificial tears to improve tear film stability and interventions targeting MGD to enhance lipid secretion and glandular function [5]. However, not all available treatments effectively address both tear film instability and MGD, highlighting the need for formulations that provide better lubrication while supporting ocular surface homeostasis [6]. One proposed therapy is hydroxypropyl-guar (HP)-Guar nano-emulsion artificial tears, which contain a polymer that enhances tear film stability by forming a protective network on the ocular surface [7]. This formulation is designed to improve lubrication and prolong tear retention time, potentially benefiting individuals with DED [8]. Given that alterations in meibomian gland structure and function lead to increased tear evaporation and instability, this formulation aims to compensate for the deficient lipid layer by improving tear retention and reducing evaporation. However, despite these proposed advantages, its effects on symptom relief and objective clinical parameters, specifically in EDE patients, have not been established.

This study aims to evaluate the effects of a 90-day treatment using HP-Guar nano-emulsion artificial tears on symptoms and signs in individuals with mild-to-moderate EDE. The study was structured across three main objectives: (1) assessing changes in DED symptomatology and quality of life, (2) assessing the evolution of MGD severity based on the structural status of lid margin features and meibomian glands and (3) assessing the progression of EDE-related parameters that impact tear film stability and ocular surface health.

## Methods

### Study Design

The study was designed as a single-masked, longitudinal clinical trial without a control group or placebo. Participants were aware of the treatment, while examiners performing the evaluations were blinded to the treatment phase and the results of previous visits. Sample size estimation was performed prior to the study using PS Power and Sample Size Calculations Version 3.1.2 (Copyright © by William D. Dupont and Walton D. Plummer, spowerandsamplesize.org/ps/). The calculation was based exclusively on an expected pre-post difference of 6.5 points in the Ocular Surface Disease Index (OSDI) score, a standard deviation (SD) of 15.1 points, statistical power of 80% and an anticipated dropout rate of 10% [9]. Consistent with prior literature, patient‑reported outcomes such as OSDI often require larger sample sizes than objective signs (e.g., non-invasive break-up time (NIBUT), meibometry, ocular redness) [10]; therefore, secondary and tertiary endpoints were evaluated as exploratory in this study, and non‑significant findings should be interpreted accordingly. According to these parameters, the minimum required sample size at the beginning of the study was 48 subjects.

Based on this calculation, a homogeneous group of volunteers with mild-to-moderate EDE was recruited from patients who had attended the clinic for a vision examination. All participants had been referred for a comprehensive evaluation due to a prior medical diagnosis of DED. During the initial visit, inclusion and exclusion criteria were verified and a battery of diagnostic tests was performed to confirm compatibility with an EDE diagnosis based on the Tear Film Ocular Surface Dry Eye Workshop II and III criteria [10–16]. Volunteers were included based on the following criteria: OSDI score between 13 and 32 (to exclude non-DED and neuropathic conditions), NIBUT <10 s and/or staining grade >1 on the Oxford Grading Scale, Tear Meniscus Height (TMH) >0.20 mm but <0.50 mm (to exclude aqueous-deficient dry eye (ADDE) and mixed dry eye participants), MGD grading score ≥5 points, good general health and no previous MGD treatments or treatments discontinued for at least 3 months before enrolment. Additionally, volunteers were excluded based on the following criteria: any ocular or systemic diseases affecting the study outcomes, such as previous eye surgery, glaucoma, diabetes, etc.; current contact lens wearers; current use of medication or artificial tears; allergy to fluorescein; history of corneal hypoesthesia, corneal ulcers, infiltrates or recurrent ocular infections and being pregnant or breast-feeding at the time of the study. All participants gave written informed consent, and all procedures were in accordance with the Helsinki Declaration. The study protocol was approved by the Bioethics Committee of the political region (CEImG-2023-327).

Recruited participants underwent four evaluations at 30 ± 3-day intervals: a baseline visit (day 0) before treatment, followed by three visits at 30 (±3), 60 (±3) and 90 (±3) days post-treatment [17–19]. Participants were instructed to instil 1–2 drops per eye of HP-Guar nano-emulsion (Systane Complete® - Alcon Laboratories, alcon.com) based artificial tears at least four times daily for 30 days. Monitoring of treatment compliance was achieved using a study product usage questionnaire at all follow-up visits. Moreover, investigators recorded the number of units dispensed as well as the bottle weight before and after usage to estimate the quantity of product used by all participants. Each session included three groups of standardised tests in alignment with the study objectives: (1) for assessing changes in DED symptomatology and quality of life, (2) for assessing the change in MGD severity based on the structural status of lid margin features and meibomian glands and (3) for analysing the progression of other EDE-related parameters as indicators of the tear film stability and ocular surface health.

### Evaluation of the Primary Objective: Changes in Symptomatology and Quality of Life

DED symptomatology was quantified using the OSDI questionnaire in all sessions [10]. The questionnaire was self-administered, and scores ranged from 0 to 100 points.

Quality of life was assessed using the Euro Quality of Life 5-Dimension 5-Level [EQ-5D-5L] questionnaire in the first (baseline) and last (90-day) sessions [20]. This questionnaire consisted of two parts. Results were computed as ‘total score’ (first part) and as ‘health today’ (second part).

### Evaluation of the Secondary Objective: Evolution of MGD Severity

MGD severity was assessed with the MGD grading score and the meibomian gland loss area (MGLA) [11, 21]. The images for the MGD grading score were obtained using a Topcon SL-D4 slit lamp (Topcon Corporation, topconhealthcare.eu) and the OCULUS Keratograph 5M (OCULUS GmbH, oculus.de) at all sessions [11]. For the final score, four lid margin features identified with the slit lamp and two meibomian gland characteristics observed through meibography were graded by a second masked observer using the MGD grading score [11].

MGLA was analysed using the OCULUS Keratograph 5M multidiagnostic platform combined with ImageJ v1.53t software (imagej.nih.gov/ij) at all sessions [21, 22]. Results were expressed as a percentage.

### Evaluation of the Tertiary Objective: Progression of EDE-Related Parameters

#### Lipid Layer Production and Status

The meibomian gland function was evaluated based on the meibomian gland expression (MGE), meibometry and lipid layer pattern (LLP).

MGE was assessed using the Topcon SL-D4 slit lamp [23]. The quality of the expressed secretion was classified into four grades: Grade 0 (clear), Grade 1 (cloudy), Grade 2 (granular) and Grade 3 (opaque solid).

Meibometry was performed using the Meibometer MB 560 (Courage-Khazaka electronic, courage-khazaka.com/en) [24]. The Sebumeter® foil strip (Courage-Khazaka electronic, courage-khazaka.com/en), employed for sample collection and subsequent measurement, was read three consecutive times, and the average value was used. Results were expressed in Meibometer units (MU).

LLP was captured using an EASYTEARview+ (Easytear s.r.l., easytear.it/en) interferometer attached to a Topcon SL-D4 slit lamp (Topcon Corporation, topconhealthcare.eu) [25]. To standardise the observation area across all videos, the Tearscope was fixed to the slit lamp at a consistent distance and illumination area. The extracted images were classified by a second masked observer according to Guillon’s basic LLP scheme into five grades [25, 26]: Grade 1 (Open Meshwork), Grade 2 (Closed Meshwork), Grade 3 (Wave), Grade 4 (Amorphous) and Grade 5 (Colour). These were then converted into a numerical system ranging from 1 (thinnest) to 5 (thickest) for analysis.

#### Tear Film Stability

Tear film stability was assessed using the OCULUS Keratograph 5M multidiagnostic platform [27]. The instrument automatically provided two parameters: the first break-up time (NIBUT-first), representing the time at which the first break occurred and the average break-up time (NIBUT-average), calculated as the mean of all break-up times across the corneal surface.

#### Inflammation and Ocular Damage

Ocular redness and corneal damage were assessed using the OCULUS Keratograph 5M multidiagnostic platform [28, 29]. For ocular redness, scores were reported for specific regions, including limbal-nasal, limbal-temporal, bulbar-nasal and bulbar-temporal areas. The redness level was automatically graded by the device on a five-point scale, with increments of 0.1 units. Regarding corneal damage, a second masked observer analysed the recorded videos using the Oxford Grading Scale, which classifies ocular staining into five severity grades [16]: Grade 0 (no staining), Grade 1 (mild), Grade 2 (mild-moderate), Grade 3 (moderate) and Grade 4 (severe).

### Other Tests Used as Inclusion Criteria

In addition to the tests previously described (although outside the study’s main objectives), TMH was evaluated using the OCULUS Keratograph 5M multidiagnostic platform to rule out ADDE. TMH was used solely as a classification criterion and not included as an outcome measure. Results were provided in millimetres.

### Statistical Analysis

IBM SPSS statistical software v. 25.0 for Windows (ibm.com) was used for data analysis, with a significance level set at *p* ≤ 0.05 in all tests. Prior to analysis, normality was assessed using the Shapiro–Wilk test for questionnaires and the Kolmogorov-Smirnov test for ocular surface parameters due to the sample size [30]. The OSDI, MGLA (in both the upper and lower eyelids) and NIBUT-average followed a normal distribution (Shapiro–Wilk or Kolmogorov-Smirnov test, all *p* ≥ 0.07). In contrast, the EQ-5D-5L total score, ‘Health Today’ parameter, MGD grading score, MGE, meibometry, LLP, NIBUT-first, ocular redness (bulbar, limbal and temporal bulbar), and corneal staining grade exhibited a non-normal distribution (all *p* ≤ 0.03). Descriptive statistics are presented as mean ± SD for normally distributed variables, median (25th–75th percentiles) for non-normally distributed variables and minimum and maximum values for all cases.

Differences in normally distributed continuous variables were assessed using repeated-measures ANOVA, with Sidak tests for pairwise comparisons [31]. Sphericity was assessed with Mauchly’s *W* test, and when violated (*p* ≤ 0.05), corrections based on epsilon (*ε*) (Greenhouse–Geisser or Huynh–Feldt) were applied to adjust the degrees of freedom and control for Type I error inflation [31]. Differences in non-normally distributed continuous variables were assessed using the Wilcoxon paired test for two related measures and the Friedman test for three or more. When the Friedman test indicated statistical significance, Wilcoxon paired tests with Bonferroni correction were used for pairwise comparison [32].

### Ethical Considerations

All procedures were conducted in accordance with the ethical standards of the institutional and regional research committee and with the 1964 Helsinki Declaration and its later amendments. The study protocol was approved by the Regional Bioethics Committee (CEImG-2023-327).

## Results

**Fig. 1:**
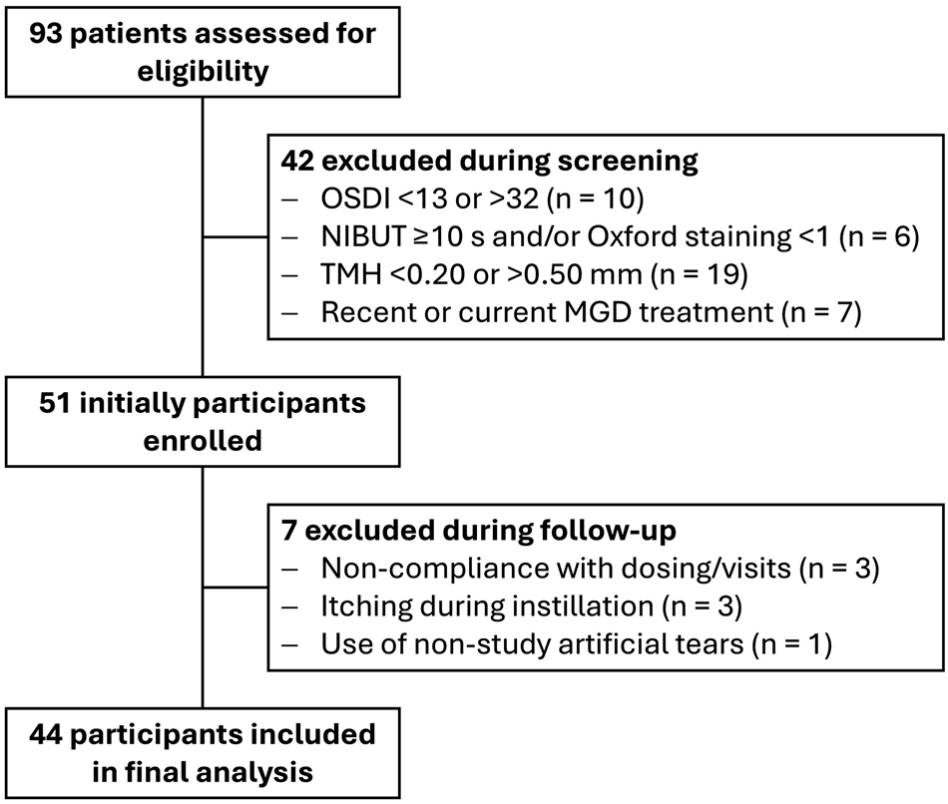
Flow diagram of participant recruitment, screening, enrolment and follow-up. OSDI Ocular Surface Disease Index, NIBUT non-invasive break-up time, TMH Tear Meniscus Height, MGD meibomian gland dysfunction.

A total of 93 patients with a previous diagnosis of EDE were initially contacted for an ocular examination in which the inclusion criteria were reviewed, and their willingness to participate was assessed. During the screening process, 42 participants were excluded for not meeting the study inclusion criteria: an OSDI score outside the predefined range (13–32) (10 participants), a NIBUT ≥10 s and/or corneal staining grade <1 on the Oxford Grading Scale (six participants), TMH <0.20 mm or >0.50 mm (19 participants) and current or recent use of MGD treatments within the 3 months prior to enrolment (seven participants). Consequently, 51 participants with mild-to-moderate EDE fulfilled all eligibility criteria and were enroled in the study. During the follow-up period, seven participants were excluded from the analysis due to protocol deviations or non-compliance: failure to adhere to the dosing or visit schedule (three participants), subjective itching during instillation without associated clinical signs (three participants) and the use of non-study artificial tears during the study period (one participant). Therefore, the final analysed sample consisted of 44 participants. Participant flow is illustrated in Fig. 1.

At baseline, all included participants exhibited TMH values within the predefined inclusion range for EDE (mean ± SD = 0.29 ± 0.08 mm; range: 0.20–0.49 mm). As TMH was used exclusively as a classification criterion and not as an outcome measure, it was descriptively reported at baseline to confirm the exclusion of ADDE and mixed dry eye phenotypes.

### Primary Objective Results: Changes in DED Symptomatology and Quality of Life

A significant difference in the OSDI score was observed across sessions (ANOVA for repeated measures [Greenhouse–Geisser adjustment], *p* = 0.001; Table 1). Pairwise analysis revealed significant differences between the baseline session and the 30-, 60- and 90-day sessions (Sidak post-hoc, all *p* ≤ 0.003), indicating an early decrease in OSDI that was sustained throughout the follow-up period. No significant differences were found between the other sessions in pairwise comparisons (Sidak post-hoc, all *p* ≥ 0.50; Table 1). Regarding the EQ-5D-5L, pairwise analysis revealed no significant differences between baseline and 90-day sessions in the total score (Wilcoxon test, *p* = 0.17; Table 1), whereas significant differences were found for the ‘Health Today’ parameter (Wilcoxon test, *p* = 0.03; Table 1). Because participants were unmasked, improvements in OSDI and the EQ‑5D‑5L ‘Health Today’ scale should be interpreted considering potential expectancy effects.

**Table 1:** Descriptive statistics of the questionnaires.

Parameter		Session	Mean^a^	SD^a^	Median^b^	Percentiles^b^		Min.	Max.
						25	75		
OSDI		Baseline	23.40	6.66	-	-	-	13.00	32.33
		30 days	18.42	10.47	-		-	2.08	44.08
		60 days	17.08	11.97	-	-	-	0.00	45.83
		90 days	15.87	11.75	-	-	-	0.00	42.00
EQ-5D-5L	Total score	Baseline	-	-	6.00	5.00	7.00	5.00	13.00
		90 days	-	-	6.00	5.00	7.00	5.00	13.00
	Health today	Baseline	-	-	80.00	71.25	90.00	30.00	97.00
		90 days	-	-	85.00	80.00	90.00	40.00	98.00

*n* = 44.

*SD* standard deviation, *OSDI* Ocular Surface Disease Index, *EQ-5D-5L* Euro Quality of Life 5-Dimension 5-Level.

^a^Mean and SD displayed on parametric parameters.

^b^Median and percentiles displayed on non-parametric parameters.

### Secondary Objective Results: Evolution of MGD Severity

A significant difference in the MGD grading score was observed across sessions (Friedman test, *p* < 0.001; Table 2). Pairwise analysis revealed significant differences between all sessions (Wilcoxon test with Bonferroni correction, all *p* ≤ 0.001), except between the 30- and 60-day sessions (Wilcoxon test with Bonferroni correction, *p* = 0.06) (Table 2).

**Table 2:** Descriptive statistics of the MGD grading scale and MGLA (Area %).

Parameter		Session	Mean^a^	SD^a^	Median^b^	Percentiles^b^		Min.	Max.
						25	75		
MGD grading score		Baseline	-	-	10.00	8.00	12.00	5.00	24.00
		30 days	-	-	9.00	7.00	11.00	3.00	22.00
		60 days	-	-	9.00	7.00	10.75	3.00	21.00
		90 days	-	-	8.00	6.00	10.00	3.00	15.00
MGLA (Area%)	Superior	Baseline	27.53	12.40	-	-	-	5.58	65.99
		30 days	28.49	13.47	-	-	-	4.88	74.02
		60 days	29.49	13.09	-	-	-	1.00	68.77
		90 days	28.25	11.99	-	-	-	8.54	73.59
	Inferior	Baseline	42.95	12.95	-	-	-	23.52	75.07
		30 days	42.09	13.42	-	-	-	19.15	76.51
		60 days	41.04	13.22	-	-	-	12.40	70.92
		90 days	38.03	12.98	-	-	-	16.25	74.59

*n* = 88.

*SD* standard deviation, *MGD* meibomian gland dysfunction, *MGLA* meibomian gland loss area.

^a^Mean and SD displayed on parametric parameters.

^b^Median and percentiles displayed on non-parametric parameters.

Additionally, a significant difference was found in MGLA in the lower eyelid across sessions (ANOVA for repeated measures [Greenhouse–Geisser adjustment], *p* < 0.001; Table 2). Pairwise analysis revealed significant differences between the 90-day session and both the baseline and 30-day sessions (Sidak post-hoc, both *p* ≤ 0.004), while no differences were observed between the other sessions (Sidak post-hoc, all *p* ≥ 0.07) (Table 2). On the other hand, no significant overall differences were found in MGLA in the upper eyelid across sessions (Friedman test or ANOVA for repeated measures [Greenhouse–Geisser adjustment], all *p* = 0.28) (Table 2).

### Tertiary Objective Results: Progression of EDE-Related Parameters

A significant difference was observed in LLP across sessions (Friedman test, *p* = 0.001; Table 3). Pairwise analysis showed significant differences in LLP between the baseline session and both the 60- and 90-day sessions (Wilcoxon test with Bonferroni correction, *p* = 0.02; Table 3). No significant differences were observed between the remaining sessions (Wilcoxon test with Bonferroni correction, all *p* ≥ 0.08; Table 3).

**Table 3:** Descriptive statistics of MGE, Meibometry, LLP, NIBUT, Bulbar redness, Limbal redness and Corneal staining.

Parameter		Session	Mean^a^	SD^a^	Median^b^	Percentiles^b^		Min.	Max.
						25	75		
MGE		Baseline	-	-	0.00	0.00	1.00	0.00	2.00
		30 days	-	-	0.00	0.00	1.00	0.00	2.00
		60 days	-	-	0.00	0.00	0.00	0.00	3.00
		90 days	-	-	0.00	0.00	1.00	0.00	2.00
Meibometry (MU)		Baseline	-	-	450.5	338.0	513.3	89.0	672.0
		30 days	-	-	397.0	272.0	508.5	129.0	693.0
		60 days	-	-	429.0	324.0	511.0	113.0	727.0
		90 days	-	-	385.0	290.0	480.5	123.0	596.0
LLP		Baseline	-	-	2.00	1.00	2.00	1.00	5.00
		30 days	-	-	2.00	1.00	2.00	1.00	5.00
		60 days	-	-	2.00	2.00	3.00	1.00	5.00
		90 days	-	-	2.00	2.00	3.00	1.00	5.00
NIBUT (s)	First	Baseline	-	-	9.55	5.18	13.56	2.23	24.92
		30 days	-	-	8.09	4.64	13.37	2.55	24.00
		60 days	-	-	8.66	5.28	14.78	2.99	24.00
		90 days	-	-	8.76	5.61	13.54	2.87	24.07
	Average	Baseline	12.80	6.05	-	-	-	2.23	24.92
		30 days	11.87	5.57	-	-	-	2.87	24.00
		60 days	12.91	5.84	-	-	-	3.97	24.00
		90 days	13.03	5.30	-	-	-	3.25	24.07
Bulbar redness	Temporal	Baseline	-	-	0.94	0.70	1.27	0.27	2.37
		30 days	-	-	0.92	0.71	1.43	0.23	2.80
		60 days	-	-	0.90	0.70	1.28	0.20	2.20
		90 days	-	-	0.97	0.70	1.32	0.40	1.93
	Nasal	Baseline	-	-	1.29	0.97	1.59	0.53	3.87
		30 days	-	-	1.25	0.97	1.67	0.30	2.97
		60 days	-	-	1.22	0.93	1.59	0.30	2.30
		90 days	-	-	1.25	1.00	1.67	0.30	2.80
Limbal redness	Temporal	Baseline	-	-	0.53	0.33	0.80	0.00	1.57
		30 days	-	-	0.57	0.40	0.90	0.00	2.00
		60 days	-	-	0.57	0.33	0.82	0.00	1.50
		90 days	-	-	0.59	0.40	0.80	0.10	1.40
	Nasal	Baseline	-	-	0.57	0.40	0.87	0.00	1.60
		30 days	-	-	0.67	0.47	0.96	0.13	1.90
		60 days	-	-	0.63	0.43	0.82	0.00	1.87
		90 days	-	-	0.70	0.43	0.92	0.10	2.00
Corneal staining		Baseline	-	-	0.00	0.00	1.00	0.00	3.00
		30 days	-	-	0.00	0.00	1.00	0.00	3.00
		60 days	-	-	0.00	0.00	1.00	0.00	3.00
		90 days	-	-	0.00	0.00	1.00	0.00	3.00

*n* = 88.

*SD* standard deviation, *LLP* lipid layer pattern, *MGE* meibomian gland expression, *MU* Meibometer units, *NIBUT* non-invasive tear break-up time.

^a^Mean and SD displayed on parametric parameters.

^b^Median and percentiles displayed on non-parametric parameters.

However, no significant overall differences were found for MGE, meibometry, NIBUT-first, NIBUT-average, limbal or bulbar redness in the temporal or nasal areas or corneal staining across sessions (Friedman test or ANOVA for repeated measures [Greenhouse–Geisser adjustment], all *p* ≥ 0.09) (Table 3).

## Discussion

The most common form of DED, EDE, is primarily linked to alterations in meibomian gland structure and function, as well as ocular surface health. These changes inherently disrupt tear film stability by increasing evaporation, leading to associated symptomatology and a potential impact on the quality of life. Current treatments focus on symptom relief and restoring gland function, but not all address both aspects effectively. HP-Guar nano-emulsion artificial tears have been proposed to enhance lubrication and tear retention, though their efficacy in EDE remains unclear. This study evaluated the effects of a 90-day treatment with HP-Guar nano-emulsion tears on symptoms, MGD severity and tear film stability in mild-to-moderate EDE patients. The present study provides new insights into the effects of HP-Guar nano-emulsion artificial tears on participants with mild-to-moderate EDE.

The findings indicated that after 90 days of treatment, participants experienced a reduction in DED symptomatology, with a parallel improvement in perceived health status. Previous reports have also found a decrease in DED symptomology with the use of a similar formulation [23,33]. This improvement suggests that the formulation reduces ocular discomfort. Furthermore, the observed enhancement in quality-of-life scores underlines the clinical relevance of symptom relief in individuals with EDE, reaffirming the necessity of therapeutic strategies that address both subjective and objective disease parameters. Nevertheless, given the single‑masked design (masked assessors, unmasked participants), changes in OSDI and EQ‑5D‑5L may be partially influenced by non‑specific study effects and participant expectations. Therefore, these person‑reported outcomes should be interpreted with caution and confirmation in double‑masked, controlled trials is warranted. Consistent with previous reports, DED symptoms were associated with higher scores in general quality of life, suggesting the need to adopt appropriate health practices and measures in this field to optimise individuals’ overall functioning and well-being [34]. A short-term study lasting 4 weeks has confirmed these findings, particularly in moderate MGD patients treated with an HP-Guar-based formulation [23]. Further research is needed to determine whether longer‑term use enhances patient satisfaction and quality of life [35]. In this context, the present study suggests that the regular use of tear drops may reduce this symptomatology and enhance the quality of life of individuals with EDE.

In addition to symptom improvement, the study demonstrated a significant reduction in MGD severity, as evidenced by improvements in lid margin features and meibomian gland structure. The severity reduction in these anatomical aspects suggests that prolonged use of HP-Guar nano-emulsion artificial tears may help mitigate structural deterioration, potentially preserving long-term glandular function, probably by reducing oxidative stress and inflammation due to malfunctioning meibomian glands. Previous studies have shown lipid mediators may influence inflammation and tissue health in the ocular surface, including the meibomian glands [36]. This reduction in inflammation could be attributed to the anionic phospholipids included in the nanodroplets of the artificial tears, which help to stabilise the polar lipid layer over the underlying aqueous phase [7]. In addition, by artificially restoring the lipid layer with lipid-based tear drops, these formulations might reduce the inflammatory processes and oxidative stress that contribute to the structural deterioration of the glands, which occurs in persons with EDE due to the malfunctioning of the meibomian glands [37]. Therefore, the improvement in meibomian gland structure observed in the present study may be linked to the positive impact of lipid-based treatments on both the lipid composition and the prevention of further glandular damage.

Another notable finding is the improvement in the tear film lipid layer, as indicated by an increased LLP thickness over the study duration, despite no changes in lipid production (assessed by MGE and meibometry) or functional stability (evaluated by NIBUT). Furthermore, as the sample size was determined for the primary objective (changes in DED symptomatology), analyses of NIBUT, meibometry and ocular redness may have been underpowered, which could partly account for the absence of statistical significance in these parameters. Previous studies have shown that lipid-based artificial tears impact lipid layer thickness positively in MGD participants, supporting their role in improving tear film stability [38]. In a similar direction, a study comparing a similar artificial tear composition in DED patients versus non-lipid-containing drops, under both normal and simulated adverse conditions, demonstrated superior performance of the HP-Guar-based tears in terms of tear stability and lipid layer thickness [13]. However, contrary to the present findings, Sindt and Foulks reported a slight but significant improvement in both MGE and NIBUT in MGD participants using an HP-Guar formulation [23]. Additionally, a short-term study (28 days) comparing HP-Guar tears between ADDE and EDE participants found increased tear stability in both groups, though to a slightly greater extent in EDE participants [39]. While these findings may appear contradictory, they indicate a consistent improvement in the tear film lipid layer. Given the importance of the lipid layer in maintaining tear film integrity, these results suggest that the HP-Guar formulation enhances lipid layer performance, potentially contributing to greater tear film stability. As conventional artificial tears primarily target ADDE rather than EDE, the observed lipid layer improvement underscores the potential of this nano-emulsion formulation to address lipid insufficiencies in persons with EDE. This is particularly relevant given that tear film instability in EDE leads to ocular surface inflammation and discomfort.

It is interesting to note that, although the overall MGD severity score and LLP improved significantly over the course of the study, more specific functional parameters such as MGE and meibometry remained unchanged. This apparent discrepancy might be explained by the inherent variability of these assessments or may suggest that structural improvements occur earlier than functional changes that can be detected objectively. In addition, the significant reduction in MGLA of the lower eyelid, observed after only 90 days, should be interpreted with caution. From a biological standpoint, it is unlikely that atrophied glands would regenerate within such a short timeframe solely through topical tear supplementation. Taken together, these findings point to potential limitations related to the sensitivity and temporal responsiveness of the evaluated parameters. They also highlight the need for longer‑term studies to elucidate better the relationship between gland morphology and functional outcomes in response to treatment.

Regarding ocular health, the present study found no significant changes in ocular redness or corneal damage over the study period in the mild-to-moderate EDE sample. Similar results with the present formulation regarding corneal damage have been reported when applied to subjects suffering from contact lens discomfort or over a short-term period (14 days) [40,41]. Contrary to these findings, other investigations have reported an improvement in conjunctival and corneal damage with the use of this formulation, but in moderate DED participants, without distinguishing between dry eye subtypes [33]. This suggests that the lack of changes observed in the present study may be due to a lower potential for improvement in individuals with mild-to-moderate EDE [10]. Additionally, if the treatment’s efficacy varies by DED subtype, then the lack of differentiation in previous studies could have contributed to conflicting results. Future work should compare the treatment effects between stratified patient groups based on DED severity and underlying mechanisms to understand its effectiveness better.

Although the study was exploratory, it also presents several methodological strengths that support preliminary findings. The use of a standardised and comprehensive assessment protocol ensured consistent measurement across visits, while the 90‑day longitudinal follow‑up with four assessment points allowed the observation of short‑term clinical trends. The strict TFOS‑based inclusion criteria resulted in a well‑defined and homogeneous mild‑to‑moderate EDE sample, strengthening internal validity. Examiner masking and the systematic monitoring of adherence further reduced measurement variability. Together, these features enhance the robustness of the exploratory observations and provide a solid foundation for future controlled trials.

Despite the outcomes, the study has some limitations that must be acknowledged. The absence of a placebo/control group precludes causal attribution and hampers separation of the intervention’s specific effects from natural history, regression to the mean and non‑specific study effects (e.g., Hawthorne/expectancy) [42]. Accordingly, these results are presented as exploratory rather than confirmatory. Moreover, because the study was single‑masked (masked assessors; unmasked participants), patient‑reported outcomes (OSDI, EQ‑5D‑5L) may be susceptible to expectancy bias and should be interpreted with appropriate caution. A randomised, double‑masked, controlled design would strengthen internal validity and provide more definitive evidence. Future such trials are warranted to validate the magnitude of effects observed in participant-reported outcomes. Additionally, while the study assessed multiple clinical parameters, other relevant inflammatory biomarkers which could provide further mechanistic insights into the observed improvements have not been measured. In line with this, the present study did not assess whether treatment response differed across baseline characteristics such as disease severity, age, sex or initial MGD grade. Given the sample size, subgroup analyses were not powered and were therefore not conducted. Future randomised, double‑masked, controlled trials with larger samples and pre‑specified subgroup analyses should corroborate these findings, identify patient profiles most likely to benefit from HP‑Guar nano‑emulsion therapy and clarify the mechanisms through which these drops influence meibomian gland function and ocular surface health. Moreover, the exclusion of a small number of participants due to non-compliance or protocol deviations may have affected the internal validity of the study. However, these exclusions were limited in number (7 out of 51 enroled participants) and followed predefined protocol criteria; thus reducing the potential for systematic bias. Treatment adherence was actively monitored through usage questionnaires and bottle weight measurements, which partially mitigates concerns related to compliance and supports the robustness of the reported findings.

In conclusion, over 90 days, HP-Guar nano-emulsion artificial tears were associated with improvements in patient-reported outcomes (symptoms and ‘Health Today’), MGD-related indices (composite severity, lower-lid gland loss) and LLP, whereas tear-film stability, ocular redness and corneal staining did not show significant changes. Given the single-masked design and the absence of a control group, these findings should be considered exploratory and confirmed in randomised, double-masked, controlled trials.

## Data Availability

The datasets generated and/or analysed during the current study are not publicly available due to participant confidentiality, but they are available from the corresponding author on reasonable request.
